# Patients’ experiences of using a smartphone application to increase physical activity: the SMART MOVE qualitative study in primary care

**DOI:** 10.3399/bjgp14X680989

**Published:** 2014-07-25

**Authors:** Monica Casey, Patrick S Hayes, Fergus Glynn, Gearóid ÓLaighin, David Heaney, Andrew W Murphy, Liam G Glynn

**Affiliations:** Discipline of General Practice, School of Medicine, National University of Ireland, Galway, Ireland.; Discipline of General Practice, School of Medicine, National University of Ireland, Galway, Ireland.; Graduate Entry Medical School, University of Limerick, Limerick, Ireland.; National Centre for Biomedical Engineering and Science, National University of Ireland, Galway, Ireland.; Centre for Rural Health, University of Aberdeen, Inverness, Scotland.; Discipline of General Practice, School of Medicine, National University of Ireland, Galway, Ireland.; Discipline of General Practice, School of Medicine, National University of Ireland, Galway, Ireland.

**Keywords:** exercise, health behaviour, primary health care, qualitative research, smartphone, technology

## Abstract

**Background:**

Regular physical activity is known to help prevent and treat numerous non-communicable diseases. Smartphone applications (apps) have been shown to increase physical activity in primary care but little is known regarding the views of patients using such technology or how such technology may change behaviour.

**Aim:**

To explore patients’ views and experiences of using smartphones to promote physical activity in primary care.

**Design and setting:**

This qualitative study was embedded within the SMART MOVE randomised controlled trial, which used an app (Accupedo-Pro Pedometer) to promote physical activity in three primary care centres in the west of Ireland.

**Method:**

Taped and transcribed semi-structured interviews with a purposeful sample of 12 participants formed the basis of the investigation. Framework analysis was used to analyse the data.

**Results:**

Four themes emerged from the analysis: transforming relationships with exercise; persuasive technology tools; usability; and the cascade effect. The app appeared to facilitate a sequential and synergistic process of positive change, which occurred in the relationship between the participants and their exercise behaviour; the study has termed this the ‘Know-Check-Move’ effect. Usability challenges included increased battery consumption and adjusting to carrying the smartphone on their person. There was also evidence of a cascade effect involving the families and communities of participants.

**Conclusion:**

Notwithstanding technological challenges, an app has the potential to positively transform, in a unique way, participants’ relationships with exercise. Such interventions can also have an associated cascade effect within their wider families and communities.

## INTRODUCTION

Sedentary lifestyles are becoming more prevalent in many countries across the world.^1^,^2^ Regular exercise helps prevent and treat numerous non-communicable diseases, including diabetes, coronary heart disease, stroke, colon cancer, hypertension, obesity, and depression.^1^,^3^ Exercise is also often inexpensive, accessible, and largely acceptable to patients.^4^ Although the benefits of regular physical activity have been shown to lead to a 30% reduction in the risk of premature death, the percentage of individuals who reach exercise goals remains suboptimal; 51% of males and 48% of females in the US,^3^ and 39% of males and 29% of females in the UK.^3^ There is an urgent need, therefore, for research to explore in more depth the barriers to participation in exercise.^5^

In the SMART MOVE randomised controlled trial (RCT), Accupedo-Pro Pedometer, an off-the-shelf smartphone application (app) to promote physical activity, was investigated in primary care. This article describes the qualitative evaluation of the experiences of a purposeful sample of trial participants.

The potential of smartphones to influence human behaviour is due to the strong attachment people have to their phones, particularly as the phones are capable of many uses and often tend to be carried by people wherever they go.^6^ People also have a tendency to interact with, or check, their mobile phones regularly; this repeated reviewing or ‘checking habit’ is reinforced by immediate visible information, rewards and, in some cases, entertainment.^7^ There is a paucity of data, however, in relation to patients’ views and experiences of using mobile phones and, in particular, apps for the promotion of physical activity. There is some emerging evidence that smartphones and smartphone apps are effective at promoting physical activity,^8^,^9^ but a clearer understanding of the patient perspective and the mechanisms by which these apps might promote behaviour change is required prior to any implementation on a wider scale. This qualitative study, embedded within the SMART MOVE RCT aimed to:

- examine factors that promote and inhibit the effectiveness of smartphones in the promotion of physical activity in primary care; and
- explore the views and experiences of participants who use this technology.

### SMART MOVE trial overview

SMART MOVE was an open-label RCT of a smartphone app to promote physical activity in primary care, and formed part of an international telemedicine implementation project funded by the European Union’s Northern Periphery Programme.^10^ This study took place in the west of Ireland; participants were recruited and followed up through three primary care centres that make up the North Clare Primary Care Team, which is rurally based and covers an economically diverse, but predominantly Caucasian, population of approximately 8000 individuals.^11^ The full study protocol has been published.^12^ The trial intervention is summarised in Box 1.

**How this fits in**

Although regular physical activity is known to help prevent and treat numerous non-communicable diseases, sedentary lifestyles are becoming more prevalent. The SMART MOVE randomised controlled trial showed that a smartphone application (app) was effective in increasing physical activity in primary care. In the qualitative evaluation of this trial, participants described a sequential and synergistic process of positive change in their relationship with physical activity as a result of their interaction with the intervention; the study termed this the ‘Know-Check-Move’ effect. Evident motivational effects and the checking of habits that result from smartphone proximity provide evidence to support the potential of apps to positively impact on behaviour change with regard to physical activity and other lifestyle behaviours.

Box 1. **The SMART MOVE randomised controlled trial**

**Table:** 

**‘SMART MOVE’ trial, August 2012–June 2013**	
Registered with the International Standard Randomised Controlled Trials Register #ISRCTN99944116; ethical approval obtained.	
90 participants recruited by primary care health professionals or self-referred; screened by the research team for inclusion suitability.	
Inclusion criteria: Adult participants in the community >16 years of age Active Android smartphone users	Exclusion criteria: Acute psychiatric illness Pregnancy Participants unable to undertake moderate exercise
Baseline screening meeting: participants given study information, signed consent, randomised, and completed quality-of-life and mental-health score questionnaires. BMI, blood pressure, and heart rate measured. Smartphone application (app), Accupedo-Pro Pedometer, was downloaded to smartphones; step-count display not made visible.	
Week 1: All participants continued normal activity level; carried smartphone during all waking hours so the app could record baseline step count while remaining invisible to the participant.	
End of week 1: Randomisation code broken; participants assigned to control or intervention groups.	
Control group: App with step count continues to remain invisible Given information on benefits of exercise Instructed to increase physical activity with a goal of an additional 30 minutes’ walking exercise (equivalent to 10 000 steps) per day	Intervention group: App and step count made visible Given information on benefits of exercise Instructed to increase physical activity (goal of 10 000 steps per day); encouraged to use app to achieve goal
After completion of 8-week trial:	
Quality-of-life and mental-health score questionnaire administered; BMI, blood pressure, heart rate recorded. Quantitative results analysed with SPSS for primary outcome (mean difference in daily step count between baseline and 8-week follow up) and secondary outcomes. After trial completion, all control-group participants shown how to use the app.	
Qualitative evaluation carried out: interviews with purposeful sample of post-trial participants to explore their experiences within 4 weeks of finishing the trial.	

BMI = body mass index. SPSS = Statistical Package for the Social Sciences.

The trial demonstrated that an app successfully promoted physical activity in primary care, with a difference in mean improvement of 1029 (95% confidence interval [CI] 214 to 1843) steps per day (or approximately a half a mile) favouring the intervention. Rather than simply providing more evidence that tracking in any form supports behaviour change, this trial represents an important step forward in the challenging issue of physical activity promotion. The unique relationship that individuals have with their mobile devices, along with the immediacy and responsiveness they demonstrate within this relationship, suggest that longer-term motivational change may be possible. However, larger RCTs with a longer follow-up are required to examine the long-term sustainability of such improvements.

## METHOD

### Recruitment of interview participants

As all trial participants had smartphones, the study decided to interview both control and intervention groups to compare their respective experiences of using technology, as well as their changes in physical activity behaviour. There were additional questions for those participants with experience of using the app intervention.

Purposive sampling was used to select participants from both groups for interview and included a range of characteristics:

- age;
- sex;
- baseline activity levels; and
- smartphone literacy.

In total, 12 interviews were conducted. As is commonplace in qualitative research, an iterative approach was taken in order to be responsive to, and incorporate, findings from the data as they emerged.^13^ A maximum variety sample was initially aimed for but it became apparent that participants’ relationships with their smartphone was an important issue. In addition, an ethical decision was made to offer instruction on using the app to all control participants once the trial had finished; as a result some participants from the control group had experience of using the app when interviewed and could share those experiences. Subsequently, purposive sampling included representatives of these two groups.

Recruitment continued until data saturation was reached^14^ and no new themes emerged.

### Interviews

The interview questions were developed by reviewing other qualitative research that explored the use of technology, exercise promotion, and behaviour change to develop a sense of what questions might elicit the most informative answers. These were then discussed with the research team and lay smartphone users to decide what questions would most thoroughly explore the participants’ experiences. The final topic guide is outlined in Box 2.

Box 2. **Interview topic guide**

Questions 1–14 were used for both control and intervention participants, while questions 15–23 were used specifically for participants who had used the smartphone application (app).

1. Where did you hear about the study? Did you volunteer or were you recruited?
2. What was your initial reaction when you were asked to join?
3. What did you expect to get out of this physical activity study?
4. Were you looking forward to it and why? (If not, why not?)
5. How did you find carrying the smartphone all day? (Pockets?)
6. How did you feel about your steps being monitored?
7. How did you find working with the technology? Were there any technical difficulties?
8. What were the barriers to increasing physical activity?
9. How did you find trying to increase your physical activity for the whole 8 weeks?
10. Did you have any strategies to help?
11. How did you describe this study to others?
12. How did you feel about letting people around you know that you were trying to increase your physical activity?
13. What was their reaction while you were increasing physical activity?
14. What were the key factors to keep you mindful about increasing physical activity?
15. Were you comfortable using the app?
16. How often did you check the app during the day?
17. How did you react if your step count was lower than the goal of 10 000 steps per day?
18. What was the best thing about the app?
19. What improvements would you like to see to the app to improve its benefits?
20. How did you feel in yourself physically, mentally, and emotionally after the intervention period of increasing your physical activity?
21. Would you consider using social media (Facebook, Twitter etcetera) to discuss your exercising?
22. Do you see yourself continuing to use the app now that the study is over?
23. What would have made it easier or more motivating to keep it going?

The participants consented to the interviews being conducted and audio-recorded, and to anonymous quotations being used. To enhance reliability, all interviews were recorded and transcribed verbatim by an independent professional transcriber, and the audio-files and transcripts compared, in line with recommended qualitative research practice.^15^ Interviews were semi-structured and conducted by one researcher; a second researcher was also used in four of the 12 interviews for quality-control purposes

### Analysis

The five stages of the framework process (familiarisation, thematic framework identification, indexing, charting, mapping and interpretation)^16^ were followed in the examination of the qualitative data. Coding was partially conducted with another researcher from a different professional background for inter-coder reliability.^17^

To heighten reflexivity, four members of the research team, (a nurse, two GPs, and one clinical engineer) reviewed all the data and contributed to the thematic analysis.^18^ NVivo (version 10) was used to organise and code the transcripts to facilitate the analysis and comparison of relationships between the coded ideas.^19^

## RESULTS

In total, 14 participants were approached and 12 (86%) agreed to be interviewed. Participant characteristics are shown in Table 1. Interviewees were similar to trial participants in age, gender, baseline activity, clinical characteristics, and smartphone literacy.

**Table 1.** Characteristics of interviewed patients

**Characteristic**	***n* (%)^a^**
Mean age, years (range)	42 (17–62)

Female	9 (75)

Intervention	8 (67)

**Baseline activity**	
Daily step count, mean (SD)	4044 (3280)

**Smartphone literacy**	
Email on phone	10 (83)
Downloaded apps previously	4 (33)

**Clinical characteristics**	
Systolic blood pressure, mean (SD)	129 (16)
Diastolic blood pressure, mean (SD)	84 (8)
BMI, mean (SD)	30 (6)

a Unless otherwise stated. BMI = body mass index. SD = standard deviation.

The key themes that emerged from the data were classified into two major interrelated themes:

- transforming relationships with exercise; and
- persuasive technology tools.

There were two minor themes:

- usability; and
- cascade effect.

### Transforming relationships with exercise: the ‘Know-Check-Move’ effect

The first major theme that emerged from the qualitative interview data was transforming relationships with exercise. A clear process of change seemed to occur in the relationship between the participant and their exercise behaviour, mediated through their use of the technology; in this case, the app intervention.

**Figure 1.**
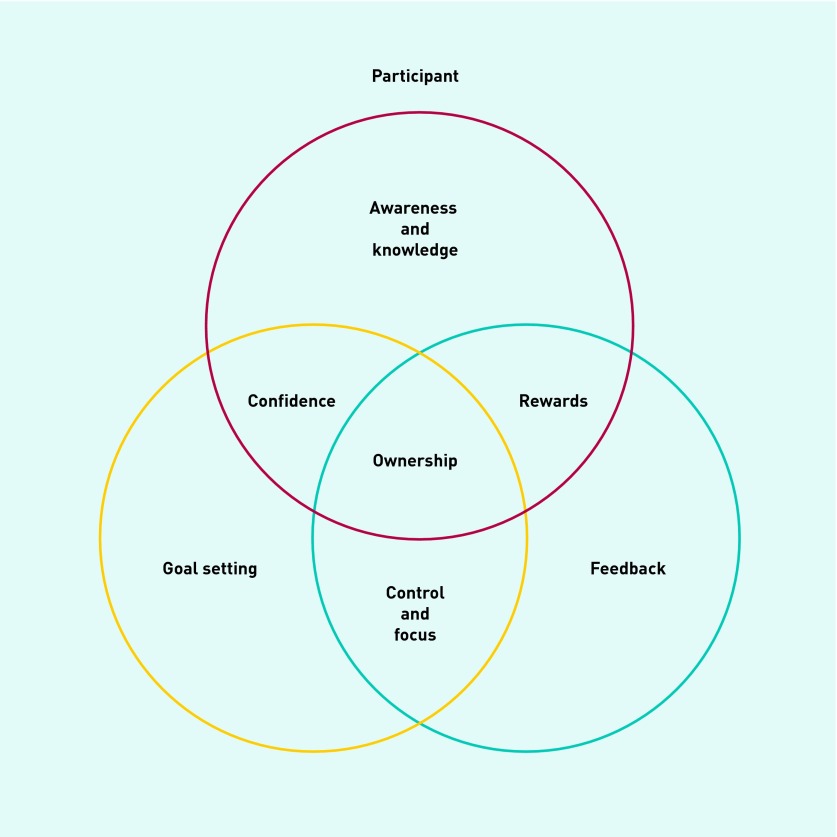
***The ‘Know-Check-Move’ effect.***

After detailed examination, the data revealed clear building blocks, sequentially structured and acting synergistically, which appeared to have the potential to create positive behavioural change around exercise. The current authors termed this novel finding the ‘Know-Check-Move’ effect, which describes how smartphone technology could affect exercise behaviour change. It consists of the following steps that became apparent in the transformational process and are interlinked:

1. Awareness and knowledge;
2. Goal setting;
3. Feedback;
4. Rewards;
5. Control and focus;
6. Confidence; and
7. Ownership (Figure 1).

#### Awareness and knowledge

Participants learned how their smartphone, when carried, could monitor their physical activity. They were curious about the number of steps completed during their usual routines and compared these with the recommended daily 10 000-step goal:

‘I never had a method for calculating the number of steps that I did before that, so I was just interested to learn what was my baseline starting off and to just register the number of steps that I did and then I could set targets and just improve my overall fitness.’

(Participant 8)

Participants were often surprised by their own daily step counts. A significant next step for them was to integrate this increased exercise into their lifestyle; as such, the app’s step-counting function served as a ‘teacher’ or tool to instruct the participants as to the actual distance required:

‘OK so once I got over the initial palpitations and had my plan, I tried to get to it and it was a focus and I found that very motivating to have that focus, that I knew now this is actually what you need to have a healthy lifestyle… you need to have this much exercise. I realised what length of a walk was going to get me my steps, you know, and what I needed to do.’

(Participant 11)

#### Goal setting

Baseline activity levels were ascertained by the participant carrying the smartphone with the application installed but invisible to them. The concept of a daily goal gave participants a focus to work towards while contemplating when, where, and how to achieve it. The technology appeared to make this process more interesting and motivating:

‘I did notice that, what I suspected, was that when I had a goal there I was far more likely to actually get up and do something.’

(Participant 2)

‘I think the goal thing was brilliant for me. Obviously I work better when I am aiming towards something.’

(Participant 3)

#### Feedback

Those assigned to the intervention group were encouraged to interact with the app, which provided live feedback on daily step count. They were given a physical activity goal of 10 000 steps per day to achieve. Those assigned to the control group were given a physical activity goal of an additional 30 minutes’ walking per day (the equivalent of 10 000 steps), along with their normal activity but did not receive any feedback on their goal attainment.

Participants using the app reported that the instant feedback put them under pressure as they checked it often and it even caused frustration if they didn’t reach the goal:

‘Because you’ve got the instant feedback to see how you get on, so that’s how it was different because it had a focus. It just tuned you in and it got you into the type of the pattern and the routine of having the focus and … well it was much easier to get into a pattern or routine with this focus and getting the feedback, and getting “yes I achieved a target today” or “I’m nearly there”.’

(Participant 11)

#### Rewards

The extrinsic reward of visualising the step count and comparing it with the 10 000 daily-step goal yielded reports of participants feeling satisfaction and a sense of achievement at their success or, alternatively, feeling frustrated if they didn’t reach their goal:

‘It’s a very easy concept, 10 000 steps a day. It’s easy to remember the phone, is easy to look at your app see how many steps you’re doing, where you are in relation to, like, the graph — you know, whether you’re at zero or near to the 10 000. It’s motivating; you want to get nearer up …you know …to move your little point along the band to get closer to the 10 000.’

(Participant 4)

Participants clearly described the motivation they experienced by using the app:

‘It’s like a “little boss in my pocket”… that’s sort of saying “you know you need to get out and do this”. I don’t know how I’d be if I turned it off. I won’t turn it off, not for the moment anyway. I am quite happy to have it on because I am much more conscious of going out for that walk.’

(Participant 9)

#### Control and focus

Different achievement strategies demonstrated how the app indirectly encouraged participants to change their exercise behaviour. With barriers such as inclement weather or dark evenings, child minding, and restrictions due to work activities, they developed alternative methods of gaining steps, such as skipping, using a cross-trainer, and walking around the house at night in order to achieve the goal. This highlighted their focus on, and control over, how to accomplish the 10 000 daily steps by timing exercise to suit their individual daily patterns:

*‘So it motivated me to find people* [babysitters] *just for that couple of minutes to get out and do the steps.’*

(Participant 4)

‘I tried to make sure that I reached certain little goals in the step count throughout the day. So by 10 o’clock I was used to have been walking a kilometre or 2km into work and by 12 o’clock I was used to having another 2000 steps done …so I tried to set little targets throughout the day so if I was tired by evening time that I’d still have accumulated most of the steps for the day.’

(Participant 8)

#### Confidence

Participants gained confidence by monitoring feedback on their activity. They began to determine how to plan exercise into their daily routine, such as averaging 2 days’ step count when they were not going to reach their daily target or working towards an overall weekly average:

‘I wanted to get the overall figure of 10 000, irrespective of some days it would have went to 12 000 and some days it would have been 8000. But there was one particular day I was driving all day and the function I was at, there was no walking involved, and then I drove all the way back, so I said that’s going to pull me down on my average.’

(Participant 10)

#### Ownership

Achieving 10 000 daily steps became a personal goal for participants, an achievement over which they felt ownership. They also reported that being able to use the app independently was an advantage, as was the fact that it was portable and could, therefore, be used anywhere:

‘If I’m going somewhere, number one you bring it with you, number two, you “park away” as I call it, and you walk further, no big deal.’

(Participant 10)

‘But you have control of this yourself. And you can just do it privately and nobody need know anything about it. And you can motivate yourself with yourself and nobody else.’

(Participant 12)

### Persuasive technology tools

The second major and interrelated theme was persuasive technology tools, as these were what emerged as the underlying mechanisms of the transformation process in the first main theme. These tools have been previously described in the persuasive technology literature and include:

- reduction;
- tailoring;
- suggestion;
- self-monitoring; and
- conditioning.^20^

All of these were evident in the data collected from participants.^20^ In addition, the proximity of the smartphone was another important factor that was identified.

#### Reduction

Reduction, or simplifying a task to influence behaviour,^20^ was evident by the reports that the app was easy to use, required basic numerical literacy, and was highly visible on the home screen. No searching was required unless participants wanted to tap the icon and access the graphs for comparisons with their step-count data from the previous day or week:

‘It was real easy you just put it in your pocket and off you go and… you could do it at your own pace.’

(Participant 5)

#### Tailoring

Tailoring is providing information specific to an individual in order to elicit a behaviour or attitude change.^20^ The step count was individual to the participant and served as a motivating factor to increase their walking exercise where possible:

‘I hated going out in the dark but I have gone out as far as the road and back to get my steps and there’s a stepper upstairs and I went on that a few times. Halfway through it, when I became more organised, I decided I know the best time for me to go do this walk is in the morning.’

(Participant 9)

#### Suggestion

**Figure 2.**
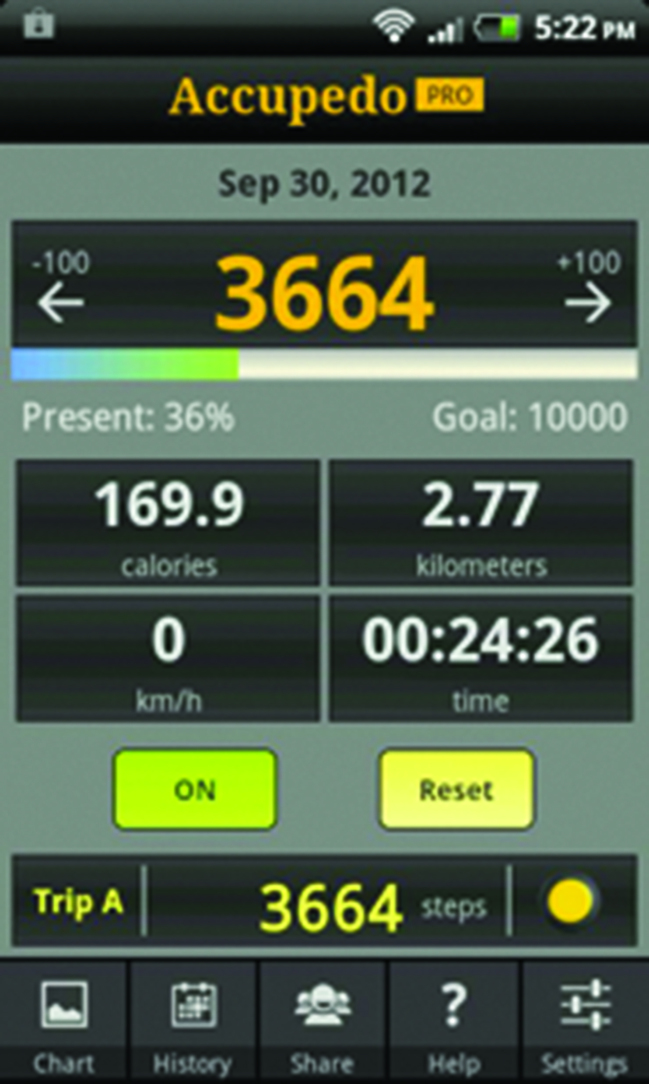
***Live step-count display of the smartphone application.***

Suggestion describes providing a cue to perform the behaviour at an opportune time.^20^ The live step-count feedback (Figure 2), which was the centrepoint of a highly visible icon on the app, made participants aware that their steps were being continually tracked, which supported a constant awareness of their goal:

‘I used to make a conscious effort not to check it up ’til lunchtime because I didn’t want to be disappointing myself because I could be standing for long periods when I was (working) and then I’d check it at lunchtime so I’d know in the afternoon I need to make up an extra conscious effort to whizz around the place. Certainly from lunchtime onwards I’d probably check it more and then, last thing, when I was leaving work I’d check it so I’d know exactly what I’d have to do in the evening time.’

(Participant 3)

#### Self-monitoring

Self-monitoring technology helps people to track their progress to change behaviour to attain a goal.^20^ Participants experienced various degrees of reported step-count checking behaviour, ranging from every few days, daily, a couple of times per day, and hourly to every time they looked at their phone:

*‘I got the app to go out in the front* [on the smartphone home screen] *so every time I opened the phone I could see the step count.’*

(Participant 5)

#### Conditioning

Conditioning technology uses rewards or positive reinforcements in order to change behaviour.^20^ The positive reinforcement of seeing feedback on their progress conditioned participants towards the achievement of their goals. Equally, when the participant viewed a low step count, this served as motivation to increase their effort:

‘You’re all day thinking well I haven’t done my 10 000 steps, how can I get another 500 up in the next half an hour or 2 hours before I go home and have to face into coming out and doing another? It was easy to get up in the morning at 6 o’clock that once or twice I did it, and maybe have 7000 or 8000 steps done, and you do 3000 steps throughout the day no problem.’

(Participant 7)

#### Proximity

Some participants reported that they always have their smartphone in close proximity, while others had to develop the new habit of carrying the phone on them at all times:

‘Very easy because I always have my phone with me anyway.’

(Participant 4)

‘I didn’t carry it on me so it was just to have it on me. When I was working I’d always leave it on the counter and when it would ring I’d come back to it so I just had to remember to put it in my pocket.’

(Participant 5)

### Usability

As with any new technology, usability challenges did arise. Some participants required assistance with downloading and setting up the app, while others encountered problems with carrying the phone, resetting the app in the mornings, and increased battery consumption; in some cases this required recharging within a 24-hour period. From a total of 90 trial participants who consented to using SMART MOVE, six discontinued due to excess battery drainage:

‘I got over some of the problems by charging it in the car but, overall, it was frustrating to try and keep it charged all the time.’

(Participant 10)

‘It was very informative. It was worth it, but carrying the phone with me was just… you had to get used to it.’

(Participant 11)

‘Well the app is very straightforward anyway so the only thing I found was I did have to reset it in the mornings.’

(Participant 1)

Features that supported usability were:

- the application worked autonomously in the background for most smartphone types;
- simple numerical literacy required; and
- large graphical display.

The importance of placing the icon on the home screen, where it could always be seen easily and not avoided, was frequently cited:

‘It is set so that when you open your phone it’s on the home screen. So you see without having to go into any app.’

(Participant 12)

### Cascade effect

A cascade effect is defined as an unforeseen series of events that can occur due to an action (participant interaction with the app) affecting a particular environment (participants’ wider lifestyle, as well as their family, social circle, and broader community). Discussion of participants’ experiences in the trial appeared to arouse curiosity and encouraged others to become aware of, and monitor, their own physical activity levels:

‘I told my family, I told the people that I was sharing a house with, and there was generally a lot of curiosity about it. I live with two other guys and one of them downloaded an app as well to start monitoring exercise.’

(Participant 8)

In certain cases this went as far as leading to an increase in physical activity levels:

‘You know because Dad, he’d never exercised so he actually got very into this idea of having some regular exercise, of going for a walk because normally his knee would be quite sore. So basically he found he was feeling better, his knee with a little bit more exercise, he was feeling better and he was getting out for it and he decided he’d take the plunge and he bought a cross-trainer.’

(Participant 11)

In addition, using the app to promote physical activity raised awareness of implementing additional positive lifestyle changes for some participants:

*‘I think I just said I’d take it as, I suppose, start from day one — try and start as you mean to go on. So I tried to drink more* [fluids]*, I changed from regular tea to decaf, I brought fruit to work so I’d snack on fruit and things like that so, overall, and I’ve continued to do it.’*

(Participant 3)

## DISCUSSION

### Summary

The data from this interview study describes a sequential and synergistic process of positive change that occurred in the relationship between the participants and their exercise behaviour; what the current authors termed the Know-Check-Move effect. Principles of persuasive technology were evident as the mechanisms that facilitated this process of behaviour change.

Evident motivational effects and checking habits that result from smartphone proximity attest to the potential of apps to support behaviour change in physical activity and other lifestyle behaviours. Not surprisingly, there were usability challenges with the technology; these included increased battery consumption and participants adjusting to always having the phone on their person.

There was also a cascade effect in participants’ wider lifestyle behaviour and among their families and communities as a result of their use of the app.

### Strengths and limitations

This study provides original data on patients’ views and experiences of using an innovative technology to promote physical activity in primary care and supports the results of the SMART MOVE trial.^9^ Other strengths of this study include the number of interviews conducted (*n* = 12) and the fact that participants used the app on their personal mobile phones. This was felt to be a pragmatic and ‘real-life’ exploration of the use of this technology in a realistic setting, and it should improve external validity of the study data. However, only smartphone users were included in the trial and, despite rapid penetration of this technology, further work is required to widen the external validity of these findings.

Data saturation was achieved during the interview process and one-on-one interviewing allowed the participants to express their individual experience unhindered by the presence of others. Reflexivity was heightened by the multidisciplinary research team reviewing the data; however, this could also be seen as a limitation as the team may have taken a different emphasis from that of an independent observer. In addition, as participants could self-refer to the study, this could have generated a response bias. Another limitation was that only Android smartphone users were included in the trial .

However due to the novel nature of the technology, it can provide a neutral space in which a clinician and patient can discuss and negotiate a management plan around the often thorny issue of sedentary lifestyle and obesity.

### Comparison with existing literature

The app evaluated in this study has some similarities to previous pedometer-based interventions that have been shown to be effective in the promotion of physical activity.^21^ However, smartphone-based interventions have the immediate advantage of requiring the user to carry only their own smartphone and not an extra piece of technology. In addition, a single, small observational study reported that participants testing three different apps aimed at promoting physical activity placed importance on extra functionality, such as automatic tracking of physical activity, goal setting, and enhanced visual feedback.^22^ This clearly resonates with the findings of the current study, in which these characteristics were clearly described.

Employing theories applicable to health-behaviour change can support efforts to harness technologies to promote physical activity,^23^ but investigation of the interactions between users and such persuasive technologies requires much greater elucidation. This is further challenged by the rapid rate of development of these technologies, which makes it very difficult for an emerging evidence base to keep pace. Larger longitudinal studies with different groups of participants would allow further exploration of the long-term effectiveness of this intervention and whether the use of this technology, like many other interventions, would wane with time.^24^ However, due to the continuous and ongoing relationship individuals have with their mobile phones, this is perhaps less likely to occur than with other technologies.

### Implications for practice

Offering exercise advice is recommended as part of routine primary care^25^ and such brief interventions have been shown to be effective in producing moderate increases in physical activity.^26^ The app evaluated here could therefore, be included in a menu of options provided by primary care health professionals for patients considering behaviour change in relation to physical activity.

Accupedo-Pro Pedometer is readily available for purchase on the open market and a version with reduced capability is available free of charge. However, health professionals may require some familiarity with smartphone apps to assist those who are considering such an intervention. As smartphone penetration and use of apps increases, which continues to happen at a very rapid pace,^27^,^28^ this will become less important. Yet, due to possible time constraints with healthcare staff in particular (as follow-up monitoring and goal adjustment may be required to achieve the best motivational effects for patients using this technology), it does remain a critical issue.

This qualitative work yields further questions regarding long-term integration of technology into primary care and patients’ daily lives, which should also be explored in future studies.

Further data with longer follow up using the app from the SMART MOVE trial is currently being conducted across six European countries.^10^ This study has demonstrated that smartphone apps have the potential to trigger a complex yet engaging and, by and large, successful behavioural-change process in relation to physical activity. This readily available motivational technology can enable individuals to take responsibility for their personal physical activity programme at a time and place that suits their schedules and lives.
